# Evidence for a possible extinction debt in Swiss wetland specialist plants

**DOI:** 10.1002/ece3.5980

**Published:** 2020-01-22

**Authors:** Anine Jamin, Markus Peintinger, Urs Gimmi, Rolf Holderegger, Ariel Bergamini

**Affiliations:** ^1^ WSL Swiss Federal Research Institute Birmensdorf Switzerland; ^2^ Department of Environmental Systems Science ETH Zürich Zürich Switzerland; ^3^ Kanton Zürich Amt für Landschaft und Natur, Fachstelle Naturschutz Zürich Switzerland

**Keywords:** bryophytes, extinction debt, habitat area, habitat fragmentation, nature conservation, vascular plants, wetlands

## Abstract

1. Habitat loss leading to smaller patch sizes and decreasing connectivity is a major threat to global biodiversity. While some species vanish immediately after a change in habitat conditions, others show delayed extinction, that is, an extinction debt. In case of an extinction debt, the current species richness is higher than expected under present habitat conditions.
2. We investigated wetlands of the canton of Zürich in the lowlands of Eastern Switzerland where a wetland loss of 90% over the last 150 years occurred. We related current species richness to current and past patch area and connectivity (in 1850, 1900, 1950, and 2000). We compared current with predicted species richness in wetlands with a substantial loss in patch area based on the species‐area relationship of wetlands without substantial loss in patch area and studied relationships between the richness of different species groups and current and historical area and connectivity of wetland patches.
3. We found evidence of a possible extinction debt for long‐lived wetland specialist vascular plants: in wetlands, which substantially lost patch area, current species richness of long‐lived specialist vascular plants was higher than would have been expected based on current patch area. Additionally and besides current wetland area, historical area also explained current species richness of these species in a substantial and significant way. No evidence for an extinction debt in bryophytes was found.
4. The possible unpaid extinction debt in the wetlands of the canton of Zürich is an appeal to nature conservation, which has the possibility to prevent likely future extinctions of species through specific conservation measures. In particular, a further reduction in wetlands must be prevented and restoration measures must be taken to increase the number of wetlands.

## Graphical abstract

We found several lines of evidence for a possible unpaid extinction debt for vascular plants in the wetlands of the canton of Zurich, that is, historic wetland area is still reflected in today's wetland specialist species richness but not in the species richness of generalist species or bryophytes. Specialist species which are in the focus of nature conservation management may thus vanish from wetlands in the future. Increasing the area of wetlands should be a main focus of the nature conservation agencies to prevent future extinctions.

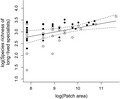

## 1 INTRODUCTION

Habitat loss, caused, for example, by land‐use change or urbanization, is the main threat to global biodiversity (Foley et al., 2005; Haddad et al., 2015; Newbold et al., 2015). Habitat loss also leads to the fragmentation of habitats into smaller patches and decreases connectivity among remaining patches (Fahrig, 2003). Small population sizes and lower gene flow are direct consequences (Ewers & Didham, 2006; Honnay, Jacquemyn, Bossuyt, & Hermy, 2005; MacArthur & Wilson, 1967). Ultimately, habitat loss and fragmentation may cause species extinction and a decline in local species richness (Noh, Echeverría, Pauchard, & Cuenca, 2019; Olsen, Evju, & Endrestøl, 2018).

According to metapopulation theory, the long‐term survival of a species in a network of habitat patches is determined by the number, size, and spatial configuration of habitat patches (Hanski & Ovaskainen, 2002). As long as the network fulfills necessary conditions in terms of habitat amount and connectivity, a species can persist in the network. If the conditions are not fulfilled, the species cannot survive and becomes eventually extinct (Hanski & Ovaskainen, 2002). However, as not all species respond at the same pace to changing environmental conditions or if the conditions are only slightly below the extinction threshold, it often takes time before a species becomes locally extinct (Hanski & Ovaskainen, 2002; Kuussaari et al., 2009). Translated to the community scale, this suggests that the number of species occurring in a habitat patch after an environmental change (e.g., decrease in habitat area or quality) will only reach a new equilibrium after some time. This delayed extinction of species is called extinction debt (Kuussaari et al., 2009). At the community level, an extinction debt can thus be characterized as a current species richness that is higher than would be expected given the present environmental conditions or area of a habitat patch (Kuussaari et al., 2009). Whether a species goes immediately locally extinct because of a change in habitat conditions or whether it is affected by an extinction debt depends on its biological characteristics (Hanski & Ovaskainen, 2002; Hylander & Ehrlén, 2013; Kuussaari et al., 2009; Saar, Takkis, Pärtel, & Helm, 2012). Specialist species are especially prone to extinction debts, because they are more dependent on a particular habitat type, and they respond sensitively to changes in environmental conditions (Henle, Davies, Kleyer, Margules, & Settele, 2004; Olsen et al., 2018). Due to the sedentariness and longevity of most plants, it can be further assumed that they are more susceptible to extinction debts than short‐lived mobile organisms such as invertebrates (Krauss et al., 2010; Morris et al., 2008; Thomas et al., 2004). It has been shown that short‐lived plants respond faster to changes in patch area and connectivity than long‐lived plants (Lindborg, 2007). Hence, long‐lived plant species and their populations can persist for extended time periods even under unfavorable environmental conditions (Bagaria, Rodà, Clotet, Míguez, & Pino, 2017; Eriksson, 1996; Krauss et al., 2010). The distribution of long‐lived plants is thus expected to be more strongly related to historical patch area and connectivity than in short‐lived plants (Kuussaari et al., 2009; Lindborg, 2007).

One of the challenges when studying extinction debt is the availability of appropriate historical and current data about habitats and species occurrence. Depending on which kind of data is available, there are different methods to identify potential extinction debt (Kuussaari et al., 2009). Here, we related current species richness of vascular plants and bryophytes, the main primary producers in wetlands, with a series of historical and current measurements of patch area and connectivity in order to check for evidence of an extinction debt. If the current species richness in wetlands is better explained by historical than by current patch area and connectivity, an extinction debt may exist (Kuussaari et al., 2009; Semper‐Pascual et al., 2018).

The study was conducted in the wetlands of the canton of Zürich in the lowlands of Eastern Switzerland. In this region, wetlands experienced a loss in area of over 90% and a severe decline in connectivity during the last 150 years, caused by land‐use intensification and later also by urbanization (Gimmi, Lachat, & Bürgi, 2011). Nowadays, the remaining wetlands have an island‐like distribution. Due to this severe habitat loss (Gimmi, Wiedmer, Graf, & Marti, 2015) and the dominance of perennial plant species in wetlands (Ellenberg, 1996), we expected the occurrence of an extinction debt. Most studies on extinction debts have been conducted in dry grasslands (e.g., Adriaens, Honnay, & Hermy, 2006; Bagaria et al., 2017; Cousins, Ohlson, & Eriksson, 2007; Helm, Hanski, & Pärtel, 2006; Lindborg, 2007; Olsen et al., 2018) or in woodlands (e.g., González‐Varo, Albaladejo, Aizen, Arroyo, & Aparicio, 2015; Kolk & Naaf, 2015; Vellend et al., 2006). Studies about the evidence of extinction debt in other habitat types such as wetlands are still rare, even though the biodiversity of wetlands has recently declined around the world (Parish et al., 2008; van Diggelen, Middleton, Bakker, Grootjans, & Wassen, 2006) and many wetland species are threatened (e.g., Bornand et al., 2016). The main causes for this decline in wetlands are drainage, peat extraction, and intensification of agriculture (Fischer, 2015; Küchler et al., 2018; Mälson, Backéus, & Rydin, 2008).

In this study, we hypothesized that (1) not only current area of wetland patches and connectivity explain current vascular plant and bryophyte species richness but also historical wetland patch area and connectivity, hence pointing to an extinction debt in the wetlands of the canton of Zürich due to severe recent habitat loss (Hanski & Ovaskainen, 2002; Kuussaari et al., 2009), (2) specialist plant species of wetlands are more likely to be affected by an extinction debt than generalist plant species, because they are more dependent on wetlands, and (3) long‐lived plant species are more likely to be affected by an extinction debt than short‐lived plant species, as long‐lived plant species respond more slowly to changing environments.

## 2 MATERIAL AND METHODS

### 2.1 Study area

The study was carried out in wetlands of the canton of Zürich (area: 1,729 km^2^, elevation range: 330–1,292 m a.s.l.) in Eastern Switzerland. The canton of Zürich has a very high population density (1.5 million; Kanton Zürich, 2019) and a sprawling urban agglomeration (Lachat et al., 2010). Despite urbanization and industrialization, numerous wetlands still exist today (total area: 12.33 km^2^) at elevations between 350 and 950 m a.s.l. (Gimmi et al., 2011). The glaciers of the Ice Age created a terrain that favored the formation of wetlands (Grünig, 2007). Even if the canton of Zürich is still rich in wetlands, especially in comparison with other regions of Switzerland, there has been a massive loss of its wetland area of more than 90% during the last 150 years due to peat extraction and drainage (Gimmi et al., 2011). The main motivation for drainage was the need to expanse the agricultural area for a growing population in the 19th and 20th century (Stuber & Bürgi, 2019). The wetlands of the canton of Zürich are nowadays well protected, and conservation management is implemented at most sites. Wetlands in the canton of Zürich can be mainly classified as fens (BUWAL, 1990). Bogs are much rarer (Grünig, Vetterli, & Wildi, 1986) and have not been considered in this study. Fens are usually mown once per year in September.

### 2.2 Wetland area and connectivity since 1850

Gimmi et al. (2011) analyzed wetland changes in terms of wetland area and connectivity in the canton of Zürich over the past 150 years, namely for 1850, 1900, 1950, and 2000. To assess the current size and distribution of wetlands in the canton of Zürich, these authors used a vectorized version of the Swiss National map for the year 2000 (swisstopo, Vector25). To reconstruct historical wetland area and connectivity in 1950 and 1900, they relied on older version of topographical maps (Sigfried maps; Gugerli & Speich, 2002). For 1850, Gimmi et al. (2011) based their reconstruction on a very detailed topographical map of the canton of Zurich (Wild map; Grosjean, 1996). All maps had a scale of 1:25,000. Because the different historical maps had used different criteria for the definition of wetlands, a complex procedure, which is explained in detail in Gimmi et al. (2011), has been applied to compare the maps. Gimmi et al. (2011) finally constructed wetland maps for all above time periods. For 1850, Gimmi et al. (2011) determined a total wetland area of about 13,759 ha in the canton of Zürich. The strongest loss of wetland area was observed between 1900 and 1950. Structural connectivity among wetlands of the canton of Zürich also declined over the last 150 years, but the greatest loss in connectivity took place during the last 50 years (Gimmi et al., 2011).

Based on the data of Gimmi et al. (2011), we first determined the area of all wetlands in 1850, 1900, 1950, and 2000, respectively. Secondly, we measured wetland area within buffers of 1km or 2km in radius with different starting points of the buffer, either from the center of a focal wetland patch or from its perimeter, to quantify present and historical connectivity. We also measured the reachable wetland area starting from the perimeter of a wetland focal patch within buffers of 1 km or 2 km in radius. In this latter case, if a wetland was positioned on the edge of the buffer not only its area strictly within the buffer but its full area, even if outside the buffer, was taken into account. In doing so, we created six connectivity variables for all wetlands in the canton of Zürich in 1850, 1900, 1950, and 2000, respectively, using arc map 10.4.1 (ESRI, 2015).

### 2.3 Plant richness of current wetlands

Presence and coverage of all vascular plant and bryophyte species were surveyed in 55 current wetlands of the canton of Zürich. The 55 wetlands were selected in a randomly stratified way out of the 708 wetland patches of the canton of Zürich in the year 2000 (Gimmi et al., 2011). Stratification criteria were current and historical patch area and connectivity. We aimed to include as much variation as possible (small/large wetlands, connected/isolated wetlands and strong/weak changes of patch area and connectivity during the last 150 years). The field survey was carried out between June 5 and August 10, 2012, by experienced wetland botanists. The survey covered all wetland (fen) types in the canton of Zürich. For orientation and precise localization of the 55 wetlands and their vegetation types, aerial photographs (at least 1:2000; map.geo.admin.ch), topographical maps (1:25’000; map.geo.admin.ch), and vegetation maps of the canton of Zürich (at least 1:2000; maps.zh.ch) were used. For data collection, at least half a day per wetland was spent searching for species. Within each wetland, all different vegetation types were covered until no new species were found. Unknown bryophytes were collected and identified later in the laboratory. The floristic data were entered into the computer program vegedaz (Küchler, 2017) to standardize nomenclature of vascular plants, which followed Lauber, Wagner, and Gygax (2012), and of bryophytes, which followed the checklist of Swiss bryophytes (Meier, Urmi, Schnyder, Bergamini, & Hofmann, 2013).

### 2.4 Species groups

We classified plant species into eight different groups: (1) all vascular plant species; (2) wetlands specialists among vascular plants; (3) generalists, which were all nonspecialist vascular plant species; (4) short‐lived vascular plant specialists; (5) long‐lived vascular plant specialists; (6) short‐lived vascular plant generalists; (7) long‐lived vascular plant generalists; (8) bryophyte species. Specialist vascular plant species included all characteristic species listed in Appendix of the wetland inventory of Switzerland (BUWAL, 1990). In the 55 wetlands studied, specialist species included mainly characteristic wetland species of the phytosociological alliances Caricion davallianae, Molinion, Magnocaricion, Phragmition, Calthion, or Filipendulion (Ellenberg, 1996). To group vascular plants into short‐ and long‐lived species, we used the plant strategy indicator of Grime, Hodgson, and Hunt (2007). It differentiates species into competitive plants (C), stress‐tolerant plants (S), and ruderal plants (R). C species are competitive and long‐lived. S species are able to survive under extreme environmental conditions and are usually long‐lived. R species are fast‐growing species with a short lifespan. Landolt et al. (2010) assigned each plant species of Switzerland to one of the following combinations: ccc, ccs, ccr, sss, css, ssr, rrr, crr, srr, csr. Species with at least one r in their three‐digit combination were classified as short‐lived, whereas species without an r were classified as long‐lived. In order to check whether this procedure made sense, we compared short‐ and long‐lived species with the mean maximum age of species in the two groups given by Landolt et al. (2010). Maximum age, however, was only available for 18.7% of the species, but the difference in mean age between the groups was significant (mean maximum age: short‐lived species: 4.7 yr ± .6 *SE*; long‐lived species: 20.8 yr ± 10.9 *SE*; one‐way ANOVA; *p*‐value = .017 in R 3.4.3; R Developement Core Team, 2017).

### 2.5 Statistical analysis

To check for collinearity among connectivity variables, we correlated them in a pairwise way using Pearson correlation coefficients for 1850, 1900, 1950, and 2000, respectively, in R. Many connectivity variables were highly correlated. We selected wetland area within a buffer of 2 km with starting point at the center of a focal wetland patch (buffer area henceforth) for our models, because it was highly correlated with all other connectivity variables in the different time periods (average correlation *r* = .83). The area of focal wetland patches (patch area henceforth) was only moderately correlated with buffer area in all time periods (*r* between .20 and .45).

**Figure 1:**
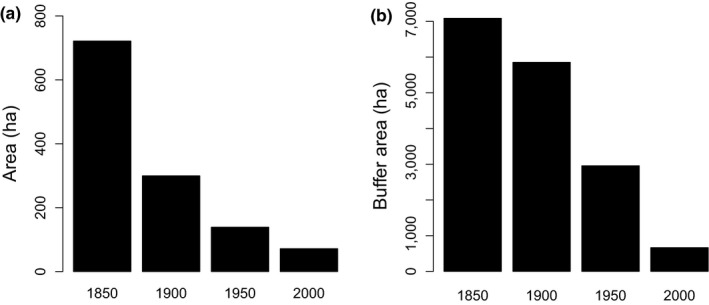
Change in total wetland area (a) and total wetland area in a buffer of 2 km around focal wetlands (b) during the last 150 years in the 55 wetlands surveyed

In absolute numbers, the patch area of the 55 focal wetlands decreased most strongly between 1850 and 1900 (Figure 1a). Proportional area loss, however, was very similar between the periods (1900:41% of the area from 1850 left; 1950:46% of the area from 1900 left; 2000:51% of the area from 1950 left). Absolute buffer area decreased strongly between 1900 and 1950 and between 1950 and 2000 (Figure 1b). Proportional loss was largest between 1950 and 2000 (77% of wetland area within the buffer lost).

We first used multiple linear regressions to analyze whether current species richness was better explained by historical or current patch area and buffer area. Species richness per wetland of each of the eight species groups was used as dependent variable and patch area and buffer area as independent variables for the four periods 1850, 1900, 1950, and 2000, respectively. We log‐transformed all dependent and independent variables. Due to outlier values, we performed robust regression using function lmrob with default settings in package robustbase (Maechler et al., 2018) in R. In robust regressions, outliers do not need to be removed, as their effects on the model are reduced giving less weight to large residuals (Rousseeuw & Leroy, 1987). For all independent variables, we additionally performed simple linear regressions and used the *R* ^2^ values to analyze their influence on current species richness.

To determine the species richness that would be expected based on a given current wetland area, we divided the wetlands into wetlands with a relatively constant area since 1850 and wetlands with a strong reduction of the area in the past, similar to Helm et al. (2006) and Piqueray et al. (2011). Constant‐area wetlands were those 27 focal wetlands with a loss in area of less than 50% during the last 150 years (average remaining area 88%). In the 28 wetlands with an area reduction in more than 50%, the average remaining area was only 18%. In the constant‐area wetlands, patch area was a significant or marginally significant predictor (*p* always below .06) for the species richness of specialists, the species richness of short‐lived specialists and the species richness of long‐lived specialists in all four time periods (Table A1). All confidence intervals of regression coefficients were considerably overlapping for each of the response variables in all four time periods, and slopes for each response variable in the four time periods differed by no more than 10.05% (*SD* 7.04%) on average. The species richness‐area relationship in these patches thus remained relatively stable, that is, species richness is consistent with an equilibrium conclusion. We therefore used the regression of current species richness on current patch area (both variables log‐transformed) in the constant‐area or equilibrium wetlands to predict species richness in the area‐reduced wetlands (Table A1). For each species group, a separate robust regression model was calculated. We then performed a sign test with the SIGN.test function in R to test whether the residuals between the current species richness and the predicted species richness of area‐reduced wetlands were more often positive than negative, that is, whether the area‐reduced wetlands had an excess of species given their area and thus exhibited an extinction debt.

To assure that constant‐area and area‐reduced wetlands did not differ in habitat quality, we calculated mean indicator values according to Landolt et al. (2010) and the standard deviation of these indicator values for each wetland based on presence–absence data of vascular plants. The latter was used as an indicator of habitat heterogeneity. We considered indicator values for temperature, light availability, moisture, acidity, nutrients, amount of humus, and soil aeration. We applied *t*‐tests and Wilcoxon‐tests to test for significant differences between the constant‐area and the area‐reduced wetlands. None of the tested indicator values (mean and standard deviation) was significantly different between the two wetland types (*p* always > .1; Table A2). Additionally, we tested in the same way for differences in elevation, patch area, and buffer area between the constant‐area and the area‐reduced wetlands. There were no significant differences (*p* always > .1; Table A2). We thus concluded that the constant‐area and the area‐reduced wetlands did not differ with respect to possible confounding factors.

## 3 RESULTS

In total, we found 567 species in the 55 wetlands studied in the canton of Zürich (Table 1). An average of 70.2 (±2.6 *SE*) vascular plant species and of 27.2 (±1.3 *SE*) bryophyte species was found per wetland. Generalist vascular plant species were slightly more frequent (37.7 ± 1.7 *SE*) than specialist vascular plant species (32.5 ± 1.4 *SE*).

**Table 1:** Species richness of the 55 wetlands studied for eight species groups. Presented are total species number and the mean number of species per wetland with standard error (*SE*)

Species group	Species number	Mean	*SE*
Vascular plant species	447	70.2	±2.6
Specialists	122	32.5	±1.4
Generalists	325	37.7	±1.7
Short‐lived specialists	32	21.8	±1.0
Long‐lived specialists	90	15.2	±.9
Short‐lived generalists	193	9.9	±.6
Long‐lived generalists	114	10.7	±.5
Bryophytes	120	27.2	±1.3

*R* ^2^‐values of multiple regression models with the independent variables patch area and buffer area for specialists were higher in all time periods than those of other species groups. Models explained up to 39% of the variation for current short‐ and long‐lived specialist species richness (Table 2). Historical models for short‐ and long‐lived specialists also had high *R* ^2^‐values, mostly above 20% (Table 2).

**Table 2:** Robust multiple linear regression models based on all 55 wetlands assessed for the periods 1850, 1900, 1950, and 2000 with patch area and buffer area as independent variables and the current species richness of eight species groups as dependent variable

Period	Species group	Patch area		Buffer area		*R* ^2^
		Estimate	*SE*	Estimate	*SE*	
2000	Vascular plant species	.080	.028**	.057	.029	.20
	Specialists	.115	.034**	.125	.048*	.32
	Generalists	.065	.038	.006	.039	.04
	Short‐lived specialists	.088	.036*	.096	.055	.19
	Long‐lived specialists	.138	.032***	.118	.035**	.39
	Short‐lived generalists	.044	.047	.039	.043	.04
	Long‐lived generalists	.126	.061*	−.061	.052	.08
	Bryophytes	.146	.063*	−.015	.070	.10
1950	Vascular plant species	.097	.027***	−.013	.037	.18
	Specialists	.128	.040**	.017	.047	.21
	Generalists	.082	.034*	−.047	.042	.08
	Short‐lived specialists	.109	.040**	−.074	.048	.13
	Long‐lived specialists	.140	.037***	.023	.042	.28
	Short‐lived generalists	.065	.039	−.026	.052	.04
	Long‐lived generalists	.122	.051*	−.158	.062*	.14
	Bryophytes	.097	.055	−.135	.066*	.10
1900	Vascular plant species	.065	.030*	−.027	.045	.10
	Specialists	.098	.033**	.013	.047	.21
	Generalists	.062	.036	−.090	.046	.07
	Short‐lived specialists	.123	.038**	−.085	.050	.20
	Long‐lived specialists	.093	.033**	.036	.050	.22
	Short‐lived generalists	.058	.043	−.088	.057	.05
	Long‐lived generalists	.094	.048	−.178	.074*	.11
	Bryophytes	.076	.051	−.125	.079	.06
1850	Vascular plant species	.057	.024*	−.022	.048	.11
	Specialists	.096	.024***	−.009	.044	.25
	Generalists	.046	.031	−.074	.055	.05
	Short‐lived specialists	.108	.027***	−.086	.050	.22
	Long‐lived specialists	.081	.025**	.053	.047	.25
	Short‐lived generalists	.031	.039	−.045	.073	.02
	Long‐lived generalists	.077	.039	−.144	.073	.08
	Bryophytes	.075	.039	−.205	.077*	.12

Notes

Estimates and standard errors for all variables and *R* ^2^ of all models are presented. All dependent and independent variables were log‐transformed.

* *p* < .05; **p < .01; ***p < .001.

Current patch area had a significant positive effect on species richness of all vascular plants and bryophytes and on the species richness of specialist species (including short‐ and long‐lived specialists). In contrast, species richness of generalists was not affected by current patch area (Tables 2 and A3). Historical patch area, however, also had strong significant and positive effects on species richness of all specialists and of short‐ and long‐lived specialists in particular. Generalists were not related to historical patch area, and only the patch areas in 1900 and in 1950 had a significant positive effect on total species richness of long‐lived generalists. Historical patch area of 1850 and 1950 had a weak positive effect on bryophyte species richness, but patch area in 1900 and 2000 were not related to bryophyte species richness.

**Figure 2:**
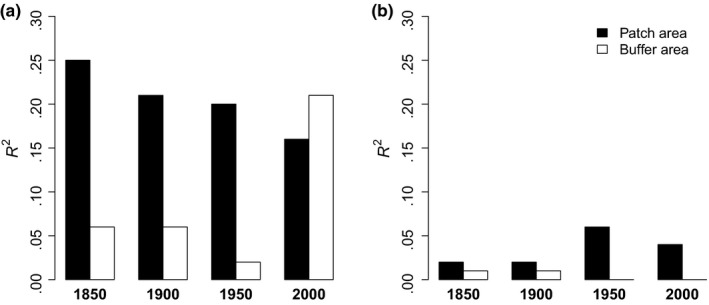
*R* ^2^ values of simple linear regressions over the last 150 years for patch area and buffer area for specialists (a) and generalists (b) wetland species

**Figure 3:**
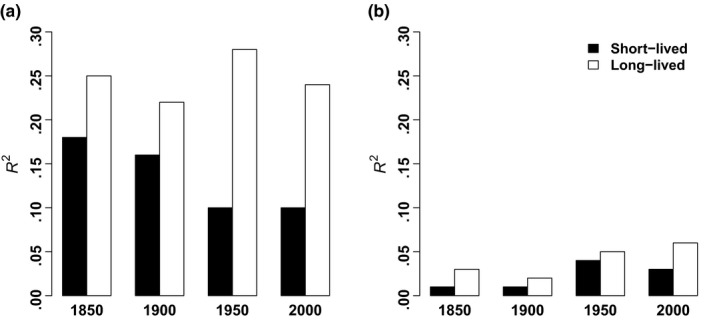
*R* ^2^ values of simple linear regressions with patch area as independent variable across the last 150 years for short‐lived and long‐lived specialist (a) and generalist (b) species

*R* ^2^‐values of single regressions of species richness against patch area were much lower for generalists than for specialists (Figures 2 and 3) and always below 10%, whereas patch area explained—in all cases—at least 16% of the variation in current specialist species richness. Patch area explained the highest amount of variation in current long‐lived specialist species in 1950, namely 28% (Figure 3; Table A3).

Buffer area was substantially less strongly related to current species richness than patch area, and the *R* ^2^‐values of the models only including buffer area were generally below 10%. Exceptions were species richness of specialists and long‐lived specialists, where current buffer area explained 21% of the variation for both groups, thus explaining a similar amount of variation than current patch area (Figure 2; Table A3). Significant positive relationships of historical buffer area in the single regressions were restricted to species richness of specialists and long‐lived specialists with buffer area of 1900 and 1850 being significant (Table A3). In the regressions including both patch area and buffer area, buffer area was positively related to species richness of specialists and long‐lived specialists, but negatively to species richness of long‐lived generalists of 1900 and 1950 and to species richness of bryophytes in 1850 and 1950 (Table 2). All other measures were not significantly affected by historical buffer area.

**Figure 4:**
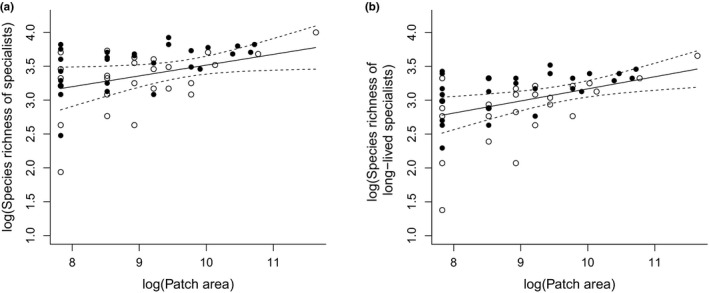
Current species richness in area‐reduced wetlands (filled circles) and constant‐area wetlands (open circles) over the last 150 years and predicted regression line (solid line) with its corresponding 95% confidence interval (dashed lines) based on the species‐area‐relationship of constant‐area wetlands for specialists (a) and long‐lived specialists (b)

Current species richness of all specialists and of long‐lived specialists in wetlands, which strongly decreased in patch area, were significantly more often above the regression line of the species‐area relationship based on constant‐area wetlands than below it (Table 3, Figure 4). This means that the species richness of specialists of wetlands that strongly decreased in patch area was higher than expected. In contrast, current species richness of short‐lived specialists, generalists, and bryophytes showed no significant effects (Table 3). Differences between the regression slopes for regressions of current species richness on the patch area of the constant‐area or of the area‐reduced wetlands were not significantly different (Table A1). However, the differences between the regression slopes (constant‐area wetland minus area‐reduced wetland) were significantly positive for the slopes of long‐ and short‐lived specialist of all periods taken together (mean difference in slopes = .065 ± .009 *SE*; *t*‐test, *p* < .001). This means that the mean slope of the regressions of specialists on patch area of the area‐reduced wetlands was shallower than the slopes of the constant‐area wetlands. For long‐ and short‐lived generalists, the difference between regression slopes (constant‐area wetland minus area‐reduced wetland) was not significant (mean difference in slopes = .005 ± .036 *SE*; *t*‐test; *p* = .205).

**Table 3:** Sign tests of the residuals between current species richness and predicted species richness based on the species‐area‐relationship of equilibrium wetlands

Species group	*p*‐value
Vascular plant species	.087
Specialists	.004**
Generalists	.087
Short‐lived specialists	.087
Long‐lived specialists	.036*
Short‐lived generalists	.572
Long‐lived generalists	.345
Bryophytes	.572

Note

*p*‐values for all species groups are presented.

* *p* < .05; **p < .01.

## 4 DISCUSSION

Our study provided several lines of evidence consistent with a possible extinction debt in the wetlands of the canton of Zürich although current habitat area and connectivity explained species richness best: (1) current species richness of wetlands with a substantial loss in area was significantly higher than expected for long‐lived specialists; (2) regression slopes for long‐ and short‐lived specialist species richness were shallower in area‐reduced wetlands than in constant‐area wetlands; (3) historical patch area and to a lesser degree also historical connectivity measured by buffer area explained significant amounts of variation in current species richness especially for long‐lived specialist species, despite low correlations between patch area and buffer area among the different time periods studied. Therefore, we assumed an unpaid extinction debt especially for long‐lived specialist vascular plant species and to a weaker degree also for short‐lived vascular specialists of wetlands. Without the implementation of adequate conservation measures, future extinctions among these species in individual wetlands are expected to occur (Kuussaari et al., 2009).

Overall, patch area explained current species richness much better than buffer area. Similar results for plants were found by Adriaens et al. (2006) and Cousins et al. (2007) in dry grasslands. While our study area had significant relationships to current species richness across all time periods (i.e., 1850, 1900, 1950, and 2000), connectivity was only rarely related to current species richness in a significant way (Table 2). Moreover, regression models including patch area had, in most cases, clearly higher *R* ^2^‐values than those with buffer area; a result further strengthening the significance of historical wetland area in explaining current species richness (Figure 2, Table A3). Interestingly, patch area in 1950 was most strongly related to current species richness in several species groups and showed the highest *R* ^2^‐values in simple linear regressions (Tables 2 and A3). Buffer area explained similar amounts of variation as area, but only when considering current data in 2000. Historical buffer area, however, explained markedly less variation than historical patch area. In other studies, habitat area and connectivity often explain similar amounts of variation (Helm et al., 2006; Noh et al., 2019), or only connectivity shows significant effects (Lindborg & Eriksson, 2004). This may be due to the fact that, in comparison to other studies, we examined three different historical time periods over a long time period of about 150 years. The conditions for finding evidence of a possible extinction debt in our study were good, as the wetland area in the canton of Zürich has declined drastically during the last 150 years, with the remaining wetland area being only about 10% of that in 1850 (Gimmi et al., 2011). Such a strong habitat loss in the past is a prerequisite for the occurrence of an extinction debt (Hanski & Ovaskainen, 2002).

The species group with the most decisive evidence for a possible extinction debt was the long‐lived specialist species of vascular plant. Current specialist species richness was significantly related to patch area in all time periods (Table 2), with the highest *R* ^2^‐value in 1950 (Figure 3; Table A3). The high *R* ^2^‐values of models of all specialists and of long‐lived specialists in particular based on historical patch area were remarkable (Figure 3; Table A3), as they generally explained between 20% and 28% of the variation in current species richness, that is, historical patch area was a good predictor of current species richness. Furthermore, the current specialist species richness of long‐lived species in wetlands that strongly decreased in patch area was significantly higher than the predicted species richness based on the species‐area relationship of equilibrium wetlands (Table 3; Figure 4). All other species groups showed nonsignificant results. Long‐lived plants are able to persist under unfavorable environmental conditions, because of their longevity. They thus build remnant populations (Bagaria et al., 2017; Eriksson, 1996). Short‐lived plants are more sensitive to and are affected more quickly by changing environmental conditions than long‐lived species. They have shorter relaxation times than long‐lived plants (Eriksson, 1996; Kuussaari et al., 2009; Schemske et al., 1994), because they have shorter generation times and must reproduce regularly in order to persist (Honnay et al., 2005; Kuussaari et al., 2009; Morriset al., 2008). However, the short‐lived specialist species also showed evidence of a possible extinction debt as historical patch area always significantly explained their current species richness. Short‐lived species may show delayed extinction in remnant habitat patches if their vital rates are only weakly affected (Hylander & Ehrlén, 2013). Furthermore, specialists are more likely to be affected by extinction than generalists as they have a higher sensitivity to changing environmental conditions. Specialists depend more strongly on particular environmental conditions and habitat types, which persist in habitat islands embedded in an unsuitable landscape matrix favoring generalist species (Adriaens et al., 2006; Ewers & Didham, 2006; Henle et al., 2004; Olsen et al., 2018). As we found stronger evidence of a possible extinction debt for long‐lived than short‐lived specialist vascular plant species in our study, all three of our hypotheses were confirmed: We found that (1) besides current patch area, past patch area also explained current species richness of vascular plants in a substantial and significant way and that current species richness was higher than expected in wetlands that lost a substantial part of their former area, (2) specialist species were more strongly affected by a possible extinction debt than generalist species and (3) long‐lived plant species were more affected by a possible extinction debt than short‐lived plant species. This greater sensitivity to an extinction debt of long‐lived specialist plant species has also been confirmed by other studies in other habitat types (e.g., Bagaria et al., 2017; Krauss et al., 2010; Lindborg, 2007; Noh et al., 2019).

The connectivity variable buffer area in 2000 showed a weaker relationship to current species richness than patch area in most species groups. However, current richness of long‐lived species and buffer area in 2000 were strongly related which points to the importance of current connectivity for these species. Functional connectivity is only strongly decreased if suitable area in a landscape is reduced to 10%–20% of its original area (Fahrig, 2003; Pardini, de Bueno, Gardner, Prado, & Metzger, 2010; With & King, 1999). In fact, such a threshold is reflected in the cover changes of the wetlands of the canton of Zürich between 1950 and 2000 (Gimmi et al., 2011) and also in our studied wetlands (Figure 1): only after the strong reduction in wetland area, connectivity declined sharply. In the simple linear regressions, historical buffer areas in 1900 and 1850 were significantly related to the species richness of specialist and long‐lived specialist species. This may point to the fact that patch area and buffer area were statistically not fully independent, although correlations were moderate between these variables in all time periods (Pearson correlation *r* between .20 and .45).

Bryophyte species richness was not affected by an extinction debt in our study: historical patch area was never significantly related to current bryophyte species richness. Current patch area, however, affected bryophyte species richness positively, although the *R* ^2^ value was low. The positive effect of current patch area and the lack of effects of historical patch area lead to the conclusion that there was no extinction debt for bryophytes. This is supported by the lack of systematic positive deviations of the species richness of area‐reduced wetlands from that of constant‐area wetlands in bryophytes. Local extinctions of bryophytes may thus have already occurred. Fast extinctions of bryophyte species in habitat remnants have been shown for epiphytes (Hylander & Weibull, 2012), and there is at least one documented example of a wetland bryophyte species (*Meesia longiseta*) that vanished from the wetlands of Zürich in the early 20th century (Hofmann et al., 2007). Although good long‐distance dispersal abilities of many bryophytes due to their small spores (Hutsemekers, Dopagne, & Vanderpoorten, 2008; Patiño & Vanderpoorten, 2018) and their capability for asexual propagation (e.g., by means of specialized propagules or clonal expansion; Rydin, 2009) should reduce the risk of local extinction in bryophytes, bryophytes are also known to react sensitively to changing environmental conditions such as changing water levels or increasing nutrients (Bergamini & Pauli, 2001; Boch et al., 2018; Vitt & Chee, 1990). Slightly changing environmental conditions in the wetlands of the canton of Zürich may thus have led to a fast payment of the extinction debt in bryophytes during the last 150 years.

Evidence for an extinction debt means that many species are not yet lost from unfavorable habitat patches, but still occur in habitat patches in which they should not actually occur due to current environmental conditions and habitat area. From our results, it can be implied that long‐lived specialist vascular plant species and, to a lesser degree, short‐lived specialists have yet only partly responded to the severe habitat loss of the wetlands of the canton of Zürich. This delayed response makes it possible to prevent or at least to reduce future extinctions of these species through specific conservation measures (Kuussaari et al., 2009; Otsu, Iijima, Nagaike, & Hoshino, 2017). The first goal must be to prevent any further reduction in wetland area. Then restoration efforts must be taken to increase the quantity of wetlands. Based on our results, restoration measures should be taken as soon as possible, as they help to remove the extinction debt and preserve, promote and protect wetland specialist species (Henle et al., 2004; Kuussaari et al., 2009). In the specific case of the wetlands of the canton of Zürich, there is no time to lose, as the reduction in habitat area of wetlands is dramatic (around 90%) and unpaid extinction debts often occur in habitat types that possess about 10% of their original area (Cousins, 2009; Gimmi et al., 2011).

## 5 CONCLUSIONS

We show that there is evidence to suggest an unpaid extinction debt in the wetlands of the canton of Zürich, especially for long‐lived specialist species and somewhat weaker for short‐lived specialists. This is evidenced by our findings that, on the one hand, the expected species richness of long‐lived specialist species in wetlands with a substantial loss in area was lower than their actually observed species richness and, on the other hand, that historical wetland area—besides current wetland area—explained a substantial and significant part of the current species richness of long‐lived wetland specialist plant species. There is still time to take conservation measures to prevent future extinction in the wetlands of canton of Zürich and elsewhere (Kuussaari et al., 2009; Mitsch & Gosselink, 2000; van Diggelen et al., 2006). Our study also confirmed that severe habitat loss leads to extinction debt of long‐lived specialist species not only in dry grasslands and woodlands (Bagaria et al., 2017; Hanski & Ovaskainen, 2002; Krauss et al., 2010) but also in wetlands. The fact that wetlands are under great pressure worldwide and rapidly decline in area (Davidson, 2014; Melton et al., 2013; Parish et al., 2008) further underlines the importance of our finding of a likely extinction debt in wetlands.

## CONFLICT OF INTEREST

None declared.

## AUTHOR CONTRIBUTIONS

A. J., A. B., and R. H. designed the study. M. P. and A. B. did the fieldwork. U. G. did the historical reconstruction of the wetlands of the canton of Zürich and calculated patch areas and connectivity measurements. A. J. and A. B. did all the analyses and wrote the manuscript, and all authors commented on it and approved the final version.

## Data Availability

Data associated with this paper are available in EnviDat: https://doi.org/10.16904/envidat.123

## Appendix 1

**Table A1:** Robust single linear regression models for the four periods 1850, 1900, 1950, and 2000 with patch area as independent variable and the species richness of eight species groups as dependent variables for the 27 constant‐area and the 28 area‐reduced wetlands studied in the canton of Zürich, Switzerland. Estimates (regression coefficients) and standard errors (*SE*) for all variables as well as *R* ^2^ values of all models are presented. Differences between estimates were tested with a *Z*‐test. None of the *Z* scores were higher than |1.96|, that is, there were no significant differences between estimates for constant‐area and area‐reduced wetlands

Period	Species group	Constant‐area wetlands			Area‐reduced wetlands			Difference between estimates	*Z* scores
		Estimate	*SE*	*R* ^2^	Estimate	*SE*	*R* ^2^		
2000	Vascular plant species	.08	.05	.07	.12	.04**	.26	−.05	−.690
	Specialists	.16	.07*	.18	.11	.04*	.20	.04	.509
	Generalists	.04	.06	.01	.11	.06	.12	−.07	−.786
	Short‐lived specialists	.14	.07*	.12	.10	.04*	.11	.04	.555
	Long‐lived specialists	.18	.06**	.29	.15	.05**	.26	.03	.402
	Short‐lived generalists	.02	.05	.00	.10	.07	.08	−.08	−.964
	Long‐lived generalists	.16	.11	.09	.09	.07	.05	.07	.559
	Bryophytes	.13	.11	.05	.13	.11	.12	.00	.007
1950	Vascular plant species	.07	.05	.07	.10	.04*	.21	−.03	−.454
	Specialists	.15	.08+	.17	.07	.05	.09	.08	.862
	Generalists	.04	.05	.01	.08	.04*	.08	−.04	−.686
	Short‐lived specialists	.14	.07+	.12	.04	.05	.02	.09	1.095
	Long‐lived specialists	.17	.06**	.27	.12	.06+	.19	.06	.622
	Short‐lived generalists	.01	.04	.00	.08	.04+	.06	−.07	−1.050
	Long‐lived generalists	.16	.11	.09	.06	.05	.03	.10	.862
	Bryophytes	.11	.11	.03	.07	.06	.05	.04	.288
1900	Vascular plant species	.07	.05	.08	.03	.03	.02	.05	.859
	Specialists	.15	.08+	.18	.07	.03*	.13	.08	1.000
	Generalists	.04	.05	.02	.00	.05	.00	.04	.551
	Short‐lived specialists	.14	.07+	.13	.08	.04*	.13	.06	.734
	Long‐lived specialists	.17	.07*	.25	.07	.03*	.12	.10	1.271
	Short‐lived generalists	.02	.04	.00	.02	.06	.00	.00	−.005
	Long‐lived generalists	.18	.10+	.13	−.01	.05	.00	.19	1.638
	Bryophytes	.11	.11	.04	.03	.05	.01	.08	.680
1850	Vascular plant species	.08	.05	.08	.03	.03	.04	.04	.791
	Specialists	.15	.08+	.18	.08	.02**	.22	.07	.943
	Generalists	.05	.05	.02	.00	.05	.00	.05	.714
	Short‐lived specialists	.14	.07*	.13	.09	.03**	.22	.05	.656
	Long‐lived specialists	.17	.07*	.25	.08	.02***	.20	.09	1.235
	Short‐lived generalists	.02	.04	.00	−.01	.06	.00	.02	.311
	Long‐lived generalists	.18	.10+	.13	.01	.05	.00	.17	1.515
	Bryophytes	.11	.11	.04	.02	.06	.00	.09	.736

^+^ *p* < .06;

* *p* < .05;

** *p* < .01;

*** *p* < .001.

**Table A2:** Indicator values (mean and standard deviation *SD* based on presence data of vascular plants) according to Landolt et al. (2010), elevation, current area, and current area in a 2 km buffer for area‐reduced and constant‐area wetlands in the canton of Zürich, Switzerland. In all cases, the differences between area‐reduced and constant‐area wetlands were not significant (*t*‐tests; *p* always > .149)

Variable	Area‐reduced wetlands	Constant‐area wetlands	*t*‐value	*p*
Temperature (mean)	3.28	3.30	−.93	.355
Light (mean)	3.34	3.29	1.22	.229
Moisture (mean)	3.73	3.76	−.52	.604
Acidity (mean)	3.28	3.24	.92	.362
Nutrients (mean)	3.02	3.04	−.30	.762
Amount of humus (mean)	3.90	3.94	−.55	.588
Soil aeration (mean)	1.33	1.33	−.01	.990
Temperature (SD)	.40	.38	1.47	.149
Light (SD)	.56	.55	.59	.556
Moisture (SD)	.71	.69	.70	.486
Acidity (SD)	.64	.65	−.09	.929
Nutrients (SD)	.78	.76	1.09	.280
Amount of humus (SD)	.99	.98	.79	.434
Soil aeration (SD)	.73	.77	−.87	.390
Elevation (m)	555	574	−.69	.492
Current area (m^2^)	8.9	9.0	−.26	.798
Current buffer area (m^2^)	11.3	11.0	1.21	.232

**Table A3:** Robust single linear regression models for the four time periods 1850, 1900, 1950, and 2000 with patch area and buffer area as independent variables and current species richness of eight species groups as dependent variables for 55 wetlands in the canton of Zürich, Switzerland. Estimates (regression coefficients) and standard errors (*SE*) for all variables as well as *R* ^2^ values of all models are presented. Dependent and independent variables were both log‐transformed

Period	Species group	Patch area			Buffer area		
		Estimate	*SE*	*R* ^2^	Estimate	*SE*	*R* ^2^
2000	Vascular plant species	.10	.03***	.14	.07	.03*	.10
	Specialists	.13	.04***	.16	.15	.05*	.21
	Generalists	.07	.04	.04	.02	.04	.00
	Short‐lived specialists	.11	.04**	.10	.12	.06	.11
	Long‐lived specialists	.16	.04***	.24	.14	.04***	.21
	Short‐lived generalists	.06	.04	.03	.05	.04	.03
	Long‐lived generalists	.11	.06	.06	−.04	.05	.01
	Bryophytes	.14	.06*	.09	.01	.07	.00
1950	Vascular plant species	.09	.03***	.18	.03	.04	.01
	Specialists	.13	.04**	.20	.05	.05	.02
	Generalists	.07	.03*	.06	−.01	.04	.00
	Short‐lived specialists	.10	.04*	.10	−.03	.05	.01
	Long‐lived specialists	.14	.04***	.28	.06	.05	.03
	Short‐lived generalists	.06	.03	.04	.00	.05	.00
	Long‐lived generalists	.09	.05	.05	−.12	.06	.06
	Bryophytes	.07	.06	.03	−.10	.07	.04
1900	Vascular plant species	.06	.02*	.09	.03	.03	.01
	Specialists	.10	.03***	.21	.09	.04*	.06
	Generalists	.04	.03	.02	−.04	.04	.01
	Short‐lived specialists	.10	.03**	.16	.01	.04	.00
	Long‐lived specialists	.10	.03***	.22	.11	.04*	.08
	Short‐lived generalists	.03	.03	.01	−.04	.04	.01
	Long‐lived generalists	.05	.05	.02	−.11	.07	.05
	Bryophytes	.04	.05	.01	−.07	.06	.02
1850	Vascular plant species	.05	.02**	.11	.04	.03	.01
	Specialists	.09	.02***	.25	.10	.05*	.06
	Generalists	.03	.03	.02	−.03	.04	.01
	Short‐lived specialists	.09	.03**	.18	.02	.05	.00
	Long‐lived specialists	.09	.02***	.25	.14	.04**	.12
	Short‐lived generalists	.02	.03	.01	−.01	.05	.00
	Long‐lived generalists	.04	.03	.03	−.07	.06	.02
	Bryophytes	.03	.04	.01	−.13	.06*	.06

* *p* < .05;

** *p* < .01;

*** *p* < .001.
